# Antitrypanosomal butanolides from *Aiouea trinervis*

**DOI:** 10.17179/excli2020-1088

**Published:** 2020-03-06

**Authors:** Felipe Oliveira Nunes, Júlio Menta de Almeida, Alda Maria Teixeira Ferreira, Letícia Alves da Cruz, Camila Mareti Bonin Jacob, Walmir Silva Garcez, Fernanda Rodrigues Garcez

**Affiliations:** 1 Instituto de Química, Universidade Federal de Mato Grosso do Sul, Av. Senador Filinto Muller 1555, 79074-460 Campo Grande-MS, Brazil; 2 Instituto de Biociências, Universidade Federal de Mato Grosso do Sul, Av. Costa e Silva s/n, 79070-900 Campo Grande-MS, Brazil

**Keywords:** Aiouea trinervis, Trypanosoma cruzi, butanolides, anti-Trypanosoma activity, in silico ADMET properties

## Abstract

In a search for new antitrypanosomal agents in the Brazilian flora, the ethanol extract of the xylopodium from *Aiouea trinervis *(Lauraceae) exhibited *in vitro* activity against the epimastigote forms of *Trypanosoma cruzi, *the etiological agent of Chagas disease. Bioassay-guided chromatographic fractionation of the ethanol extract afforded three butanolides, isoobtusilactone A (**1**), epilitsenolide C2 (**2**), and epilitsenolide C1 (**3**). Butanolides **1** and **3** were more active against *T. cruzi *epimastigotes than the reference drug benznidazole (by 8.9-fold and 3.2-fold, respectively), while **2** proved inactive. Compounds **1** and **3** showed low cytotoxicity in mammalian Vero cells (CC_50_> 156 μmol L^-1^) and high selectivity index (SI) values for epimastigotes (SI = 56.8 and 28.6, respectively), and **1** was more selective than benznidazole (SI = 46.5). Butanolide **1** at 24 μmol L^-1^ also led to cell cycle alterations in epimastigote forms, and inhibited the growth of amastigote cells in more than 70 %. *In silico* ADMET properties of **1 **were also analyzed and predicted favorable drug-like characteristics. This butanolide also complied with Lipinski's rule of five and was not predicted as interference compound (PAINS). This is the first report on the isolation of these bioactive butanolides under the guidance of *in vitro* trypanocidal activity against *T. cruzi*.

## Introduction

Chagas disease (American Trypanosomiasis), a parasitic protozoal infection caused by *Trypanosoma cruzi*, is a neglected tropical illness of high medical relevance, that affects at least six million people, mostly in the endemic regions of Latin America including Brazil, where there are currently more than 1.1 million chronically infected individuals (WHO, 2015[35]). In recent years, the number of *T. cruzi* infections acquired through congenial and unscreened blood transmissions and organ transplants have been increasing in non-endemic areas worldwide, including North America and Europe, as a result of international migration (Soriano-Arandes et al., 2016[32]). An increasing number of Chagas disease cases has also been reported in both endemic and non-endemic regions due to oral transmission via consumption of food and beverages contaminated with infected triatomine bugs and their fecal matter (Coura and Viñas, 2010[18]; Jackson et al., 201[24]4; Montgomery et al., 2014[27]).

Two currently available chemotherapies for the treatment of Chagas disease are benznidazole and nifurtimox, nitroimidazole-derived drugs that are well known for their substantial toxicity and poor efficacy during the chronic phase of the disease (Bern et al., 2007[5]). Therefore, the search for safer and more reliable trypanocidal agents, such as plant-derived secondary metabolites, is urgently needed.

*Aiouea trinervis* Meisn. is a shrub of the Lauraceae family which grows in the “Cerrado” of Mato Grosso do Sul, Brazil. The biological properties of members from this genus have been scarcely reported and refer to the evaluation of the genotoxic and/or cytotoxic potentials of only two species, namely *A. costaricensis* and *A. trinervis*. A weak cytotoxic activity against neoplastic cell lines was reported for the essential oils from the leaves and branches of *A. costaricensis* (Chaverri et al., 2010[12]), while in a previous study of the roots, xylopodium and leaves of *A. trinervis* we reported the isolation of four butanolides (epilitsenolide C1, epilitsenolide C2, isoobtusilactone A and obtusilactone A) and evaluation of the cytotoxic activities of the first three compounds against Hep2 cells (Garcez et al., 2005[22]). The genotoxicity of obtusilactone A and isoobtusilactone A isolated from *A. trinervis *was also assessed by using the somatic mutation and recombination test (SMART) on *Drosophila melanogaster* and/or the comet assay on CHOK1 and HTC mammalian cells (Garcez et al., 2005[22]; Guterres et al., 2014[23]). The ability of isoobtusilactone A obtained from *Cinnamomum kotoense *to induce apoptosis in Hep G2 cells and its apoptotic mechanisms of action were described by Chen et al. (2008[13], 2012[14]). In addition, this same compound isolated from *Persea americana *was reported to have a nematicidal potential (Dang et al., 2010[21]). Cytotoxic and anti-HIV activities have also been described for structurally similar butanolides isolated from other lauraceous species, such as *Litsea verticillata*, *L. akoensis*, *Lindera communis*, and *Cinnamomum subavenium *(Chen et al., 1998[15]; Shen et al., 2011[31]; Tsai et al., 2002[33]; Zhang et al., 2005[36]).

As part of our ongoing project aimed at the discovery of new natural product-based antitrypanosomal agents from plants occurring in the Cerrado ecosystem of midwest Brazil, we found that an EtOH extract of the xylopodium of *Aiouea trinervis *Meisn. (Lauraceae) showed *in vitro* activity against epimastigote forms of *Trypanosoma cruzi* (IC_50_ = 6.77 µg mL^-1^). Herein, we describe the isolation and structural characterization of three butanolides from this bioactive extract as well as an assessment of their *in vitro *antiprotozoal activity and cytotoxicity in mammalian Vero cells. In addition, the most active compound was evaluated against amastigote cells, for cell cycle alterations in epimastigote forms, and subjected to *in silico* prediction for ADMET properties, and detection of PAINS substructures.

## Material and Methods

### General experimental procedures

Optical rotations were determined on a Perkin Elmer 341 polarimeter. HRESIMS data were acquired on a UltrOTOF-Q instrument (Bruker Daltonics) with electrospray ionization and operating in positive mode. ^1^H and ^13^C NMR spectra were recorded at room temperature in CDCl_3_ (Cambridge Isotope Laboratories) on a Bruker DPX-300 spectrometer operating at 300.13 MHz (^1^H)/75.47 MHz (^13^C). Column chromatography procedures were performed on silica gel 60 (70-230 mesh, Merck), and Sephadex LH-20 (Amersham Biosciences). Reversed-phase semipreparative HPLC separations were carried out with a Waters 600 system using RP-18 (5 µm, 21.2 x 250 mm) in a Phenomenex Luna column at a flow rate of 11 mL min^-1^, with monitoring at 210 and 254 nm.

### Plant material

Xylopodium of *Aiouea trinervis *Meisn. was collected in Campo Grande, Mato Grosso do Sul, Brazil (20^o^31'44.6”S 54^o^24'37.1”W) in April 2016. The plant material was identified by Dr. Arnildo Pott (Instituto de Biociências, Universidade Federal de Mato Grosso do Sul). A voucher specimen (no. 37450) has been deposited at the CGMS Herbarium of the Universidade Federal de Mato Grosso do Sul.

### Extraction and isolation

Air-dried and powdered xylopodium of *A. trinervis* (265.6 g) was extracted with 95 % ethanol (EtOH) at room temperature. After concentration *in vacuo*, the bioactive EtOH extract was subsequently partitioned between methanol (MeOH)-H_2_O (9:1) and hexane, MeOH-H_2_O (1:1) and CH_2_Cl_2_, and MeOH-H_2_O (1:1) and ethyl acetate (EtOAc), to yield the respective hexane (16.5 g), CH_2_Cl_2 _(4.4 g) and EtOAc (3.4) phases.

Part of the hexane phase (16.0 g) was then chromatographed on a silica gel 70-230 mesh column (450 g, Φ: 4.5 cm, height: 48 cm), using step gradient elution with hexane, hexane-EtOAc (5 % → 50 %), and EtOAc to afford six fractions (A → F). Fraction B (7.42 g, hexane-EtOAc 8:2) was purified by column chromatography on Sephadex LH-20 (Φ: 5 cm, height: 40 cm), using hexane-CH_2_Cl_2 _(2:8), CH_2_Cl_2_-acetone (3:2) and CH_2_Cl_2_-acetone (2:3) as eluents, to give compound **1** (0.19 g). Fraction C (2.10 g, hexane-EtOAc 7:3) was chromatographed on a Sephadex LH-20 column (Φ: 3.5 cm, height: 30 cm), using hexane-CH_2_Cl_2 _(2:8), CH_2_Cl_2_-acetone (3:2) and CH_2_Cl_2_-acetone (2:3) as eluents, to afford six fractions (C_1_→C_6_). Fraction C_2_ (0.09 g, hexane-CH_2_Cl_2_ 2:8) was purified by semipreparative RP-HPLC (acetonitrile-H_2_O 88:12) to yield compounds **2** (0.075 g) and **3** (0.017 g).

Part of the CH_2_Cl_2_ phase (4.2 g) was submitted to column chromatography on silica gel 70-230 mesh (280 g, Φ: 4.5 cm, height: 29 cm) eluted with a hexane-EtOAc-MeOH gradient system (hexane, hexane-EtOAc 5 % → 50 %, EtOAc and EtOAc-MeOH 10 %) to give seven fractions (A → G). Fraction E (0.65 g, hexane-EtOAc 30 %) was subjected to gel permeation column chromatography on Sephadex LH-20 (Φ: 2.5 cm, height: 30 cm), eluted with hexane-CH_2_Cl_2 _(2:8), CH_2_Cl_2_-acetone (3:2) and CH_2_Cl_2_-acetone (2:3), followed by semipreparative RP-HPLC (acetonitrile-H_2_O 88:12), to yield compounds **2** (0.032 g) and **3** (0.012 g).

Isoobtusilactone A (**1**): Colorless oil; [α]*_D_*^23^-35 (*c* 0.416, CHCl_3_) [lit. [α]*_D_*^23^ -38 (*c* 0.425, CHCl_3_) (Garcez et al., 2005[22])]; ^1^H-NMR (300.13 MHz, CDCl_3_): *δ *7.05 (td, 1H, *J* 8.0, 2.0 Hz, H-6), 5.23 (brs, 1H, H-3), 4.92 (dd, 1H,* J* 2.8, 1.7 Hz, H-5a), 4.71 (dd, 1H, *J* 2.8, 1.4 Hz, H-5b), 2.36-2.52 (m, 2H, H-7), 1.99 (brs, 1H, OH), 1.44-1.56 (m, 2H, H-8), 1.25 (brs, 20H, H-9-H18), 0.86 (t, 3H,* J *6.8 Hz, H-19); ^13^C-NMR (75.47 MHz, CDCl_3 _): δ 166.9 (C-1),157.8 (C-4), 150.2 (C-6), 127.4 (C-2), 91.4 (C-5), 66.4 (C-3),32.0 (C-17), 29.4-29.7 (C-9-C-16), 29.6 (C-7), 28.3 (C-8), 22.8 (C-18), 14.2 (C-19); HRESIMS: *m/z* 309.2429 [M+H]^+ ^(calcd for C_19_H_33_O_3_, 309.2424); *m/z* 331.2250 [M+Na]^+ ^(calcd for C_19_H_32_O_3_Na, 331.2244).

Epilitsenolide C2 (**2**): Colorless oil. [α]*_D_*^23^ - 96 (*c* 0.26, CHCl_3_) [lit. [α]*_D_*^23^ - 97.5 (*c* 0.28, CHCl_3_) (Garcez et al., 2005[22])]; ^1^H-NMR (300.13 MHz, CDCl_3_): *δ* 6.93 (t, 1H, *J* 7.8 Hz, H-6), 4.81 (brd, 1H,* J* 5.2 Hz, H-3), 4.52 (m, 1H, H-4), 2.38 (m, 2H, H-7),1.85 (brs, 1H, OH), 1.50 (m, 2H, H-8), 1.45 (d, 3H, *J* 6.5 Hz, H-5), 1.25 (brs, 20H, H-9-H-18), 0.87 (t, 3H,* J *6.5 Hz, H-19); ^13^C-NMR (75.47 MHz, CDCl_3 _): *δ* 170.1 (C-1), 147.9 (C-6), 130.6 (C-2),78.8 (C-4), 67.8 (C-3), 32.0 (C-17), 30.0 (C-7), 29.7-29.4 (C-9-C-16), 28.5 (C-8), 22.8 (C-18), 14.2 (C-5), 14.1 (C-19); HRESIMS *m/z* 311.2585 [M+H]^+ ^(calcd for C_19_H_35_O_3_, 311.2581); *m/z *333.2401 [M+Na]^+^ (calcd for C_19_H_34_O_3_Na, 333.2401).

Epilitsenolide C1 (**3**): Colorless oil. [α]*_D_*^23^ - 78 (*c* 0.068, CHCl_3_) [lit. [α]*_D_*^23^ - 80 (*c* 0.075, CHCl_3_) (Garcez et al., 2005[22])]; ^1^H-NMR (300.13 MHz, CDCl_3_): *δ* 6.56 (t, 1H, *J* 7.7 Hz, H-6), 4.64 (brs, 1H,* J* 5.1 Hz, H-3), 4.51 (dq, 1H, * J* 6.6, 5.6 Hz, H-4), 2.74 (m, 2H, H-7), 1.80 (brs, 1H, OH),1.44 (d, 2H, *J *6.8 Hz, H-8), 1.39 (d, 3H,* J *6.5 Hz, H-5), 1.25 (brs, 20H, H-9-H-18), 0.87 (t, 3H,* J *6.5 Hz, H-19); ^13^C-NMR (75.47 MHz, CDCl_3 _): *δ* 168.7 (C-1), 149.8 (C-6), 129.4 ( C-2), 77.9 (C-4), 71.5 (C-3), 32.0 (C-17), 29.8-29.4 (C-9-16), 28.9 (C-8), 28.0 (C-7), 22.8 (C-18), 14.2 (C-5), 14.2 (C-19); HRESIMS *m/z* 311.2591 [M+H]^+ ^(calcd for C_19_H_35_O_3_, 311.2581); *m/z *333.2411 [M+Na]^+ ^(calcd for C_19_H_34_O_3_Na, 333.2401).

### Epimastigote cultures of Trypanosoma cruzi

Epimastigote forms of *T. cruzi* Dm28c were maintained in LIT (Liver Infusion Tryptose) medium, supplemented with 10 % Fetal Bovine Serum at 28 °C. Parasites in the exponential growth phase were used in the experiments.

### In vitro viability assay against epimastigotes

The effects of the extract and fractions on the viability of the epimastigote forms of the parasite were determined by a colorimetric assay using MTS/PMS. New cultures, containing 10^6^ parasites/mL, were added to the wells and incubated with the extract and fractions (at 50 µg mL^-1^) for 72 h, and the extract and fractions that showed activity were evaluated in a subsequent assay at six different concentrations (50.0-1.06 µg mL^-1^) to calculate the IC_50_. All assays were performed in technical triplicate.

After incubation for 72 h, MTS/PMS solution was added (containing 400 μg mL^-1^ and 9.2 μg mL^-1^, respectively), and the plates were incubated at 28 °C for 4 h. Then, the optical density was read at 490 nm in a microplate reader (Asys Expert Plus; Biochrom).

Parallel tests were performed using 1 % dimethylsulfoxide (DMSO, negative control) and a replicate of each concentration, and then fixed with 4 % paraformaldehyde prior to the addition of MTS/PMS solution (basal absorbance control).

### Antiproliferative activity assay of isolated compounds against epimastigotes and amastigotes

The parasites, as epimastigote forms (in exponential growth phase), were added, at 10^6^/mL, to wells containing 1 % DMSO (negative control), benznidazole (standard drug) or one of the butanolides at six different concentrations (1-80 µmol L^-1^) and incubated for 24 and 72 h. After each incubation, the parasites were counted in a Neubauer chamber to determine the antiproliferative activity. Each treatment was performed in technical triplicate and the counts were repeated in case of discrepancies between replicates. 

A growth inhibition curve for the most active butanolide (isoobtusilactone A,** 1**) and negative control was then plotted using these data. This butanolide was also analyzed for its potential activity against intracellular amastigote forms of *T. cruzi.*

For assays against intracellular amastigote forms (Dm28c), Vero cells were used as the host cells. These were cultured in DMEM medium and maintained in CO_2_ atmosphere at 37 °C. Cultures with confluence greater than 70 % were used in 96-well plates with an inoculum of 4x10^3^ cells/well with trypomastigotes (ratio 1:10). After 4 h of incubation for adherence and infection, the cells were washed with 1x PBS and then incubated in DMEM medium for 20 h for differentiation of internalized trypomastigotes into amastigotes.

Vero cells infected with amastigotes were incubated for 24 h with the most active compound (isoobtusilactone A) at a concentration of 24.0 μmol L^-1^ in DMEM medium. After incubation, the cells were washed, fixed with methanol and stained with DAPI (4',6-Diamidino-2-phenylindole), for photodocumentation under Leica DMI6000B fluorescence microscope. The photos obtained were analyzed in the software ImageJ version 1.5, for quantification of the total Vero cells, the percentage of infected cells and number of amastigotes per cell. The inhibition of amastigotes was evaluated using the infectivity index described elsewhere (Da Silva et al., 2015[20]; Ceole et al., 2018[10]). As negative control was used 1 % DMSO.

### Cell cycle analysis for flow cytometry 

Epimastigote forms were treated with isoobtusilactone A for 24 h at the concentrat- ions of 2.75 μmol L^-1^ (IC_50_/24 h) and 24.0 μmol L^-1^. Following treatment, the parasites were washed and immediately read in a FACS canto II flow cytometer (filter 585/420), after staining with propidium iodide buffer (3.4 mM Tris-HCl, 0.1 % NP40, 700U/L RNAse A, 10 mM NaCl and 7.5 µM propidium iodide).

### Cytotoxicity assay against mammalian cells

Vero cells (ATCC: CCL-81) were kept at 37 °C in a humidified 5 % CO_2_ incubator, in 25 cm^2^ culture flasks containing DMEM (Dulbecco's Modified Eagle Medium) medium pH 7.4, supplemented with 2.5 % FCS (Fetal Calf Serum), 2 mM L-glutamine, 10 μg mL^-1^ streptomycin and 10 μg mL^-1^ penicillin. Uninfected Vero cell monolayers were washed with PBS (Phosphate Buffer Saline) pH 7.2, detached by treatment with 0.25 % trypsin for 5 minutes at 37 °C, washed and resuspended in the same medium and subsequently seeded into 96-well plates (2×10^3^ cells/well). After 24 h, the cells were incubated during 24 h with the compounds at different concentrations (12 µmol L^-1^ to 400 µmol L^-1^). After treatment, the cells were replaced into new fresh medium with MTT (2 mg mL^-1^) and viability was analyzed after 4 h of incubation. The absorbance was read at 570 nm in a microplate reader (Biotek Model EL800, VT, USA). The negative (1 % DMSO) and positive (benznidazole) controls were also used in the same experiment. The negative control was performed with eight replicates, while the compounds and benznidazole were assayed with technical replicate.

### In silico analyses

*In silico* analyses of isoobtusilactone A (**1**) were conducted for prediction of some of its pharmacokinetics and toxicity - ADMET (Absorption, Distribution, Metabolism, Excretion and Toxicity) properties. These were assessed using the freely available pkCSM (http://biosig.unimelb.edu.au/pkcsm) (Pires et al., 2015[28]), Tox-Prediction (http://tox.charite.de/protox_II) (Banerjee et al., 2018[4]), STopTox (http://stoptox.labmol.com.br) (Braga et al., 2017[6]) and Pred-hERG (http://predherg.labmol.com.br) (Braga et al., 2015[7]) servers. The compliance of **1** to the Lipinski's rule of five was also calculated in the present study using the freely available pkCSM server (http://biosig.unimelb.edu.au/pkcsm) (Pires et al., 201[28]5), while analysis of pan-assay interference compounds-PAINS (exclusion of false positives) was performed using the freely available on line tool PAINS-remover (https://www.cbligand.org/PAINS) (Baell and Holloway, 2010[2]).

### Statistical analysis

All tests were analyzed using Graphpad Prism software version 7.04 for windows (GraphPad Prism, 2017, La Jolla, CA, USA). The IC_50 _and CC_50 _values were obtained by nonlinear regression with variable slope and automatic withdrawal of outliers.

## Results and Discussion

After partitioning of the bioactive EtOH extract from the xylopodium of *Aiouea trinervis*, the resulting hexane, dichloromethane and EtOAc phases were assessed for their *in vitro *effects against *Trypanosoma cruzi *epimastigotes. The results revealed that the antitrypanosomal activity resided in the hexane and dichloromethane phases, with IC_50 _values of 3.30 µgmL^-1^ and 8.18 µgmL^-1^, respectively, while the EtOAc phase was devoid of any activity. 

**Figure 1:**
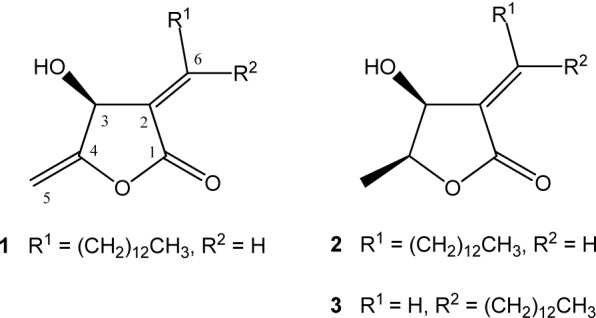
Structures of butanolides 1-3

Further trypanocidal activity-guided fractionation of the bioactive hexane and dichloromethane phases led to the isolation of the polyketide-type butanolides isoobtusilactone A (**1**) from the hexane phase and epilitsenolide C2 (**2**) and epilitsenolide C1 (**3**) from both the hexane and dichloromethane phases (Figure 1(Fig. 1)). In a previous study, we reported the isolation of butanolides **1**-**3 **from the xylopodium and roots of *Aiouea trinervis* (Garcez et al., 2005[22]). In the present investigation, the structures of **1**-**3 **were determined based on their ^1^H and ^13^C NMR, and HRESIMS data, which were in agreement with those formerly reported (Anderson et al., 1992[1]; Chen et al., 2000[16]; Garcez et al., 2005[22]; Rollinson et al., 1981[29]), as well as by comparison with authentic samples.

For this bioprospective study on anti-*T. cruzi* compounds from plant species of the Brazilian Midwest, we opted for the parasite epimastigote form (strain Dm28c), because it is a clonal population, whose axenic cultivation is well established *in vitro*, thus guaranteeing homogeneity of the assays (Camargo, 1964[9]). Therefore, the anti-trypanosomal evaluation against epimastigote forms may be employed, not only for assessment of the anti-*T. cruzi* potential of isolated compounds, but also as a screening for new promising trypanocidal agents in crude extracts. Therefore, following isolation, compounds **1**-**3 **were evaluated *in vitro* for their activity against *T. cruzi *epimastigotes, and the results are shown in Table 1(Tab. 1) (the raw data can be found in the Supplementary data, Supplementary Tables 1 and 2). 

Isoobtusilactone A (**1**) was the most active butanolide, with significant IC_50_ values, at both 24 and 72 h (2.75 and 3.32 µmol L^-1^, respectively), followed by epilitsenolide C1 (**3**) (IC_50_/24 h = 7.77 and IC_50_/72 h = 11.12 µmol L^-1)^. In contrast, epilitsenolide C2 (**2**) was inactive at the tested concentrations even after a 72 h exposure. The anti-*T. cruzi* activities of butanolides **1** and **3** were stronger than that of the reference drug benznidazole, and anti-*T. cruzi* activity of **1 **was about 9-fold higher than that of benznidazole at 24 h, and about 4-fold higher at 72 h. Similarly, albeit evidenced to a lesser degree, the activity of butanolide **3 **was approximately 68 % and 24 % higher than that of benznidazole at 24 and 72 h, respectively. 

The foregoing results revealed some possible structure-activity relationships for the isolated compounds. Based on the IC_50_ values of **2** and **3**, it can be inferred that the (*Z*)-geometry of the conjugated double bond in the α-alkylidene-γ-lactone skeleton is an important feature for the trypanocidal activity of **3**, since its corresponding (*E*)-stereoisomer **2 **showed no activity against *T. cruzi* epimastigotes. Therefore, with all other things being equal, an improvement in the activity was observed by changing the stereochemistry of the side chain from (*E*) to (Z). In contrast, butanolide **1**, which bears the same (*E*)-configuration on the side chain as **2**, but differs from the latter only by the presence of a Δ^4,5^ unsaturation, showed the strongest activity against *T. cruzi*. These results suggest that the activity of compound **1** stems from the presence of an exocyclic double bond functionality at C-4. This structural feature seems to exert a stronger effect on *T. cruzi *epimastigotes than the orientation of the side chain at C-6, since compound **1** was the most active compound, even though it has an (*E*)-configuration of the conjugated double bond. Therefore, it can be assumed that both the geometry of the conjugated double bond at C-2, and particularly the presence of an exocyclic double bond at C-4 play key roles in the activity of this class of compounds against *T. cruzi* epimastigotes.

When assessing the cytotoxicity of butanolides **1** and **3 **against mammalian Vero cells to determine their toxicity and selectivity index (SI) values, compounds **1** and **3** showed CC_50_ values >156 µmol L^-1 ^after a 24 h exposure (Table 1(Tab. 1)). The obtained SI values revealed that isoobtusilactone A and epilitsenolide C1 were respectively 56.8 and 28.6 times more selective for the epimastigote forms of *T. cruzi* than for mammalian cells. According to the Drugs for Neglected Diseases initiative (DNDi), a candidate drug for *in vivo* trials should have an SI > 10 (Chatelain, 2015[11]). It is also noteworthy that isoobtusilactone A proved more selective than benznidazole, which has an SI of 46.5.

**Figure 2:**
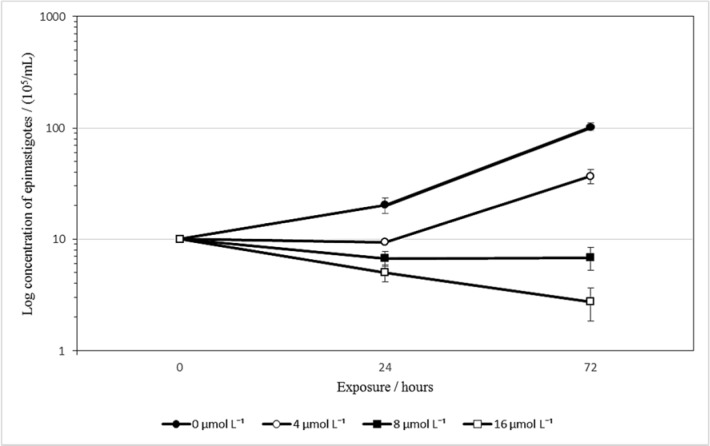
Growth curve of epimastigotes log concentration treated with isoobtusilactone A, according to the exposure time. Mean ± SD for each calculated experiment from three technical replicates

To verify the effect of isoobtusilactone A (**1**) on the multiplication of *T. cruzi *epimastigotes, their growth was analyzed in the presence of different concentrations of compound **1 **for 72 h. As depicted in Figure 2(Fig. 2), a drastic reduction in the number of parasites was observed following incubation with 8 and 16 μmol L^-1^ of isoobtusilactone A, suggesting that it has trypanocidal activity at concentrations as low as 8 μmol L^-1^. Data obtained at 72 h indicated that treatment with 4 μmol L^-1^ led to a 70 % decrease in growth when compared to the untreated control, while treatment with 16 μmol L^-1^ led to the death of more than 80 % of the parasites (the raw data of Figure 2 can be found in the Supplementary data, Supplementary Table 3).

Given the evident alteration in the growth of isoobtusilactone A-treated parasites, the percentage of epimastigotes in each phase of the cell cycle (G1, S and G2/M) was determined by flow cytometric analysis. Based on the cell growth curve data (Figure 2(Fig. 2)), two concentrations were then selected (2.75 and 24.0 μmol L^-1^). Cell cycle analysis revealed no difference between the parasites treated with the former concentration of isoobtusilactone A and control parasites, which might indicate that this compound does not affect this cellular mechanism. However, when a higher concentration of the butanolide was applied, a reduction of population in G2 phase of the cycle was observed (from 34 % to 22.3 %), as well as an increase in epimastigotes percentage in both G1 (45.5 % to 52.2 %) and S phase (18.6 % to 23.8 %). This alteration might represent a secondary damage effect caused by isoobtusilactone A to the parasite. It is worth mentioning that cell cycle arrest at the G2-M phase of cancer cell lines has been previously reported for isoobtusilactone A (Kuo et al., 2007[25]). Further studies are therefore needed so that the mechanisms of action of this butanolide can be fully clarified. 

Based on the observed cell cycle changes of epimastigote forms treated with the highest concentration of isoobtusilactone A, an intracellular replicative form growth inhibition assay was performed on the vertebrate cell host. It was shown that the same concentration able to induce cell cycle arrest of epimastigote forms also inhibited more than 70 % in growth of amastigote forms after a 24 h treatment. These data indicate the potential of this butanolide also against the replicative form of *T. cruzi* which is present in humans.

Given the foregoing results, a computational approach was conducted for prediction of some pharmacokinetics properties and toxicity (ADMET) of isoobtusilactone A (**1**)- the most active butanolide-, using the on line platforms pkCSM, Tox-prediction, STopTox and Pred-hERG. The compliance of **1** to the Lipinski's rule of five calculation, as well as pan-assay interference compounds (PAINS) analysis were also performed, using the pkCSM and PAINS-remover web servers, respectively. *In silico* estimation approaches of absorption, metabolism, excretion and toxicity (ADMET) and other drug-like properties, such as the Lipinski's rule of five and PAINS parameters, have to be considered at an early stage on evaluation of potential candidates for drug development (Baell and Nissink, 2018[3]; Lipinski et al., 2012[26]; Pires et al., 2015[28]). Therefore, according to Table 2(Tab. 2), butanolide **1** is expected to be non-carcinogenic, non-mutagenic, non-hepatotoxic, and non-hERG blocker (non-cardiotoxic), as well as having no rat toxicity by the oral route. It is also likely to be metabolized by CYP3A4 and an inhibitor of CYP2C19 enzymes, but probable neither substrate nor inhibitor of the other main cytochrome P450 isoforms analyzed. The evaluation of absorption parameters revealed that **1** has a good prediction of human intestinal permeability and absorption and a relatively low probability of skin permeability. As for blood-brain barrier (BBB) and central nervous system (CNS) permeabilities, this butanolide is predicted to have a poor CNS permeability, but a favorable BBB penetration. Compound **1** was also not recognized as a pan-assay interference compound (PAINS), which lowers the probability of its biological properties be considered as artifacts (Baell and Nissink, 2018[3]). These results, in addition to no violation of Lipinski's rule of five, support the drug-like characteristics of isoobtusilactone A.

## Conclusions

Lauraceous species are known to be sources of secondary metabolites with trypanocidal activity (Uchiyama et al., 2002[34]; Da Silva Filho et al., 2004[19]; Setzer and Setzer, 2006[30]; Cabral et al., 2010[8]; Conserva et al., 2019[17]). Among these bioactive compounds, only two butanolides (isolinderadolide D and isolinderadolide E) and a *seco*-butanolide (*seco*-subamolide A) from *Nectandra opposittifolia *have hitherto been reported (Conserva et al., 2019[17]). Although compounds **1**-**3** were obtained in our previous investigation of *Aiouea trinervis *(Garcez et al., 2005[22]), this is the first report on the isolation of the bioactive butanolides isoobtusilactone A (**1**) and epilitsenolide C1 (**3**), and the inactive epilitsenolide C2 (**2**) using *in vitro* trypanocidal activity-guided fractionation against epimastigote forms of *T. cruzi*. Given the IC_50 _and SI values of compound **1,** and its good drug-like characteristics revealed by the results obtained in the *in silico* analyses, this butanolide can be considered as a promising candidate for further investigation of its mechanisms of action against the parasitic stages of *T. cruzi,* as well as studies of structural modifications in order to potentiate its anti-*T. cruzi* properties. The bioactive butanolides from *A. trinervis* and others from the Lauraceae may thus be regarded as a promising class of compounds in the search for new trypanocidal agents.

## Acknowledgements

The authors gratefully acknowledge the Fundação de Apoio ao Desenvolvimento do Ensino, Ciência e Tecnologia do Estado de Mato Grosso do Sul (FUNDECT-grant numbers 027159 and 021683) for financial support, and the Coordenação de Aperfeiçoamento de Pessoal de Nível Superior (CAPES, Finance Code 001), for the grants awarded to F.O.N. and J.M.A. Dr. Arnildo Pott is acknowledged for his assistance in the identification of the plant material.

## Conflict of interest

The authors declare that they have no conflict of interest.

## Supplementary data

Supplementary data (^1^H and ^13^C NMR spectra and HRESIMS spectra of compounds **1**-**3**, and raw data of Table 1 and Figure 2) can be found here.

## Supplementary Material

Supplementary data

## Figures and Tables

**Table 1:**
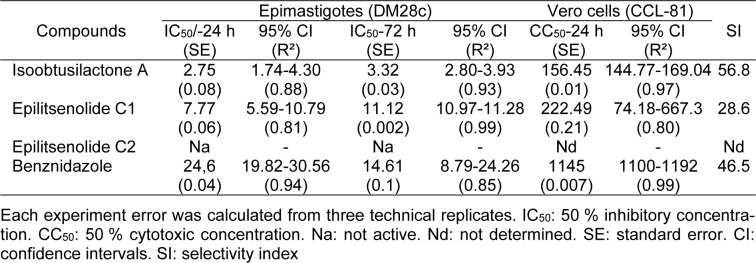
Antiproliferative activity and cytotoxicity of butanolides isolated from *A. trinervis *(μmol L^-1^)

**Table 2:**
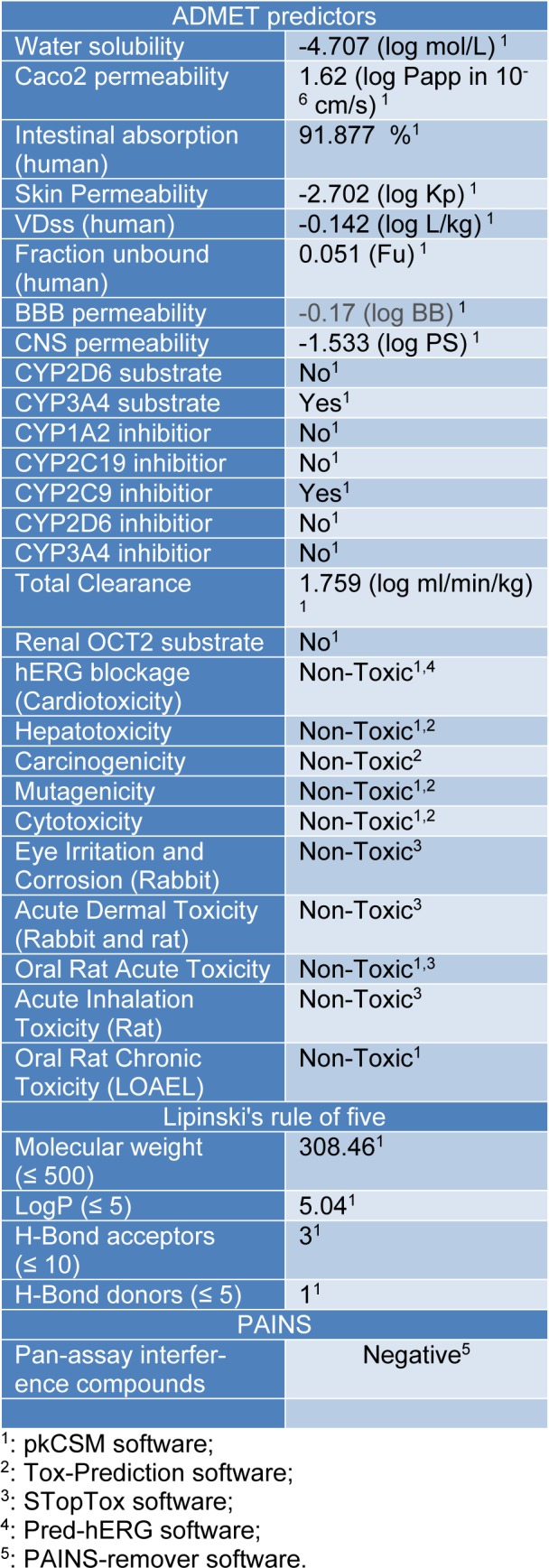
Predicted ADMET properties, Lipinski's rule of five and PAINS analyses of isoobtusilactone A.
