# Supplementary material for: Antitrypanosomal butanolides from Aiouea trinervis
Source: EXCLI J. 2020 Mar 6;19:323–33. doi: 10.17179/excli2020-1088 (PMC7174576; doi:10.17179/excli2020-1088)
Supplement: Supplementary data [file EXCLI-19-323-s-001.pdf]

EXCLI Journal 2020;19:323-333 – ISSN 1611-2156
Received: January 27, 2020, accepted: February 26, 2020, published: March 06, 2020

Supplementary data to:

ANTITRYPANOSOMAL BUTANOLIDES FROM *AIOUEA TRINERVIS*

Felipe Oliveira Nunes[1], Júlio Menta de Almeida[2], Alda Maria Teixeira Ferreira[2], Letícia Alves da Cruz[2], Camila Mareti Bonin Jacob[2], Walmir Silva Garcez[1], Fernanda Rodrigues Garcez[1,]*

[1] Instituto de Química, Universidade Federal de Mato Grosso do Sul, Av. Senador Filinto Muller 1555, 79074-460 Campo Grande-MS, Brazil
[2] Instituto de Biociências, Universidade Federal de Mato Grosso do Sul, Av. Costa e Silva s/n, 79070-900 Campo Grande-MS, Brazil

* **Corresponding author:** Fernanda Rodrigues Garcez, Instituto de Química, Universidade Federal de Mato Grosso do Sul, Av. Senador Filinto Muller 1555, 79074-460 Campo Grande-MS, Brazil. Tel.: +55-67-33453579; E-mail: fernandargarcez@gmail.com

http://dx.doi.org/10.17179/excli2020-1088

This is an Open Access article distributed under the terms of the Creative Commons Attribution License (http://creativecommons.org/licenses/by/4.0/).

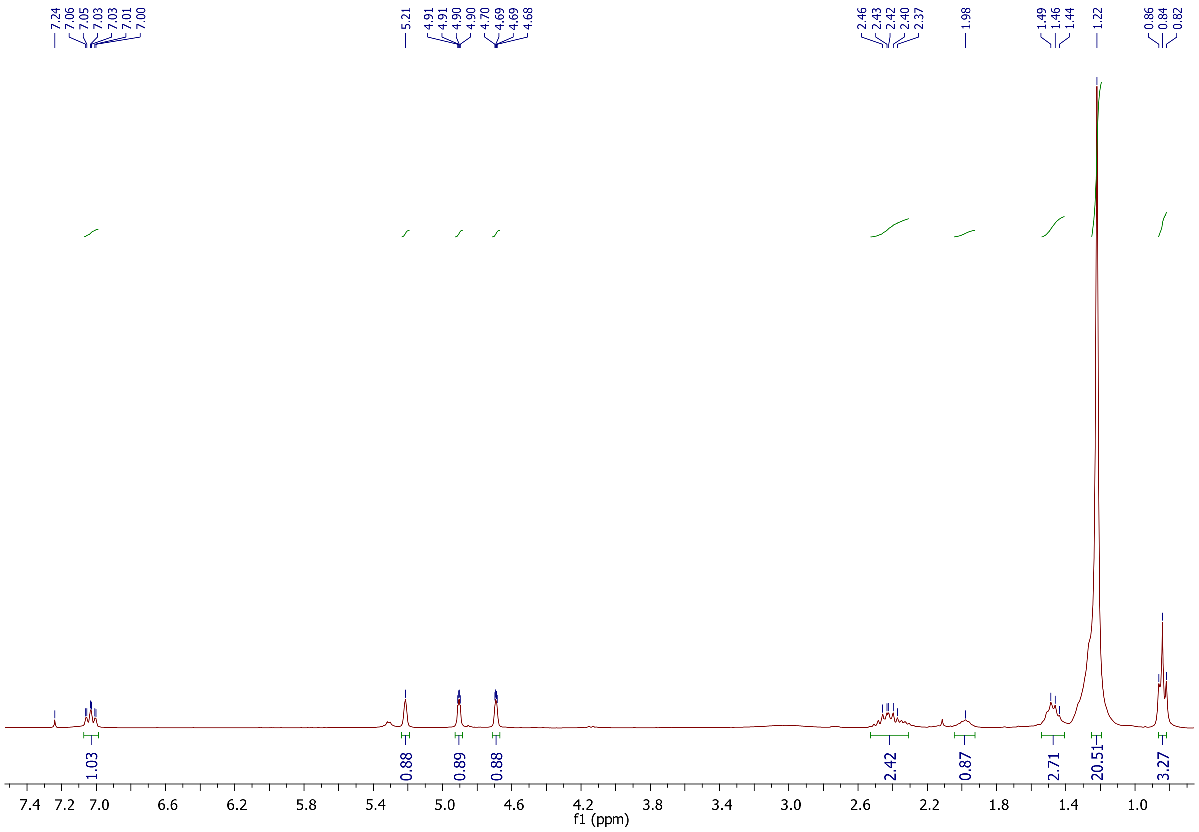

Supplementary Figure 1: ^{1}H NMR spectrum of compound **1** (300 MHz, $CDCl_3$)

EXCLI Journal 2020;19:323-333 – ISSN 1611-2156
Received: January 27, 2020, accepted: February 26, 2020, published: March 06, 2020

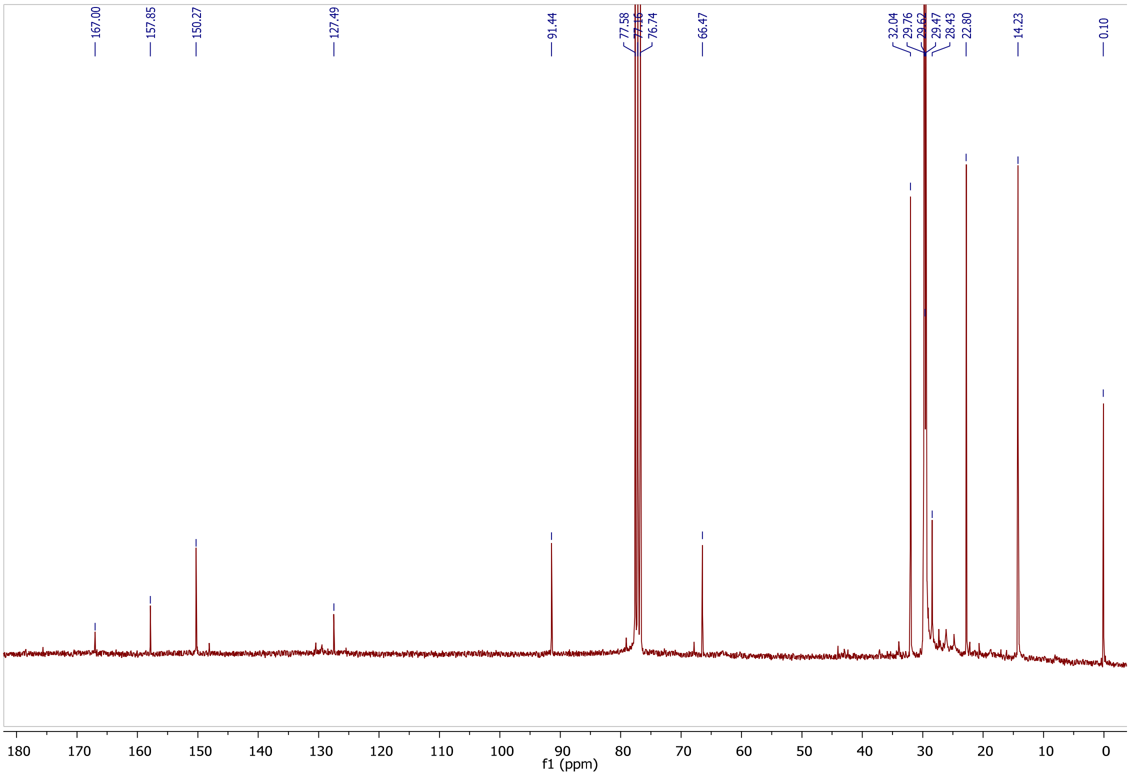

Supplementary Figure 2: ^{13}C NMR spectrum of compound **1** (75 MHz, $CDCl_3$)

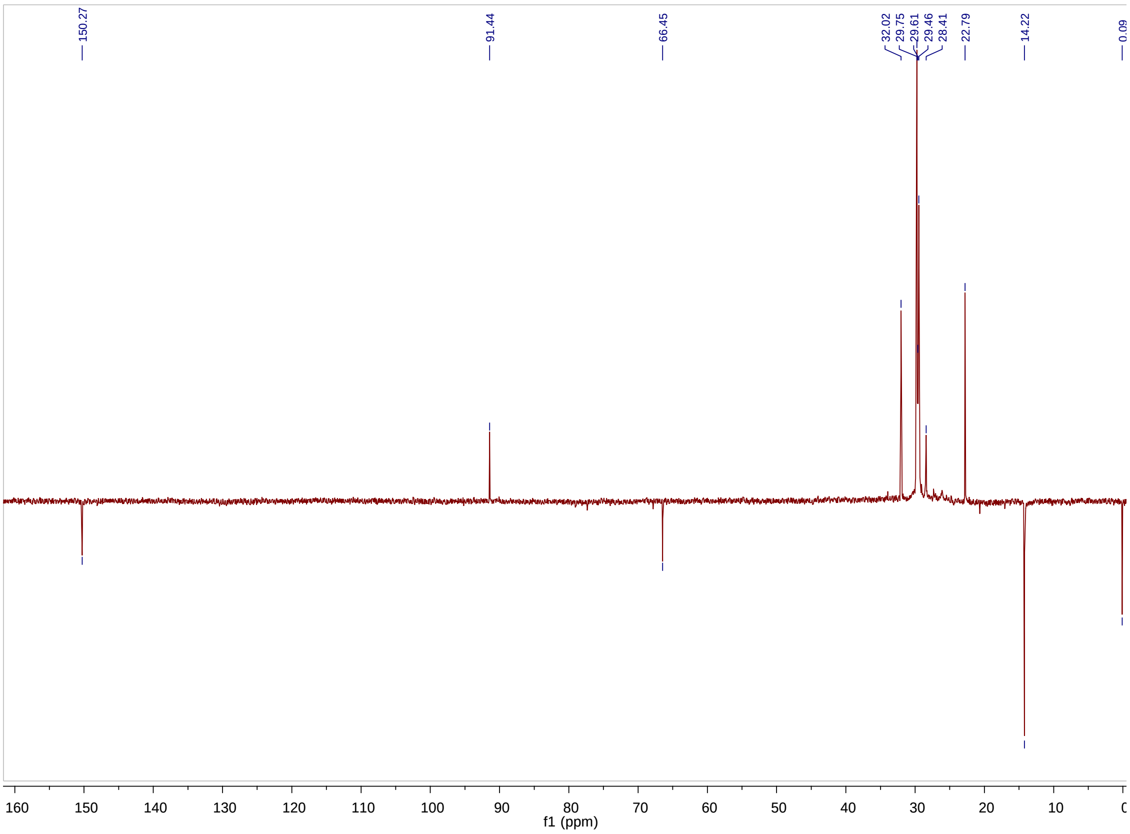

Supplementary Figure 3: DEPT-135 NMR spectrum of compound **1** (75 MHz, $CDCl_3$)

EXCLI Journal 2020;19:323-333 – ISSN 1611-2156
Received: January 27, 2020, accepted: February 26, 2020, published: March 06, 2020

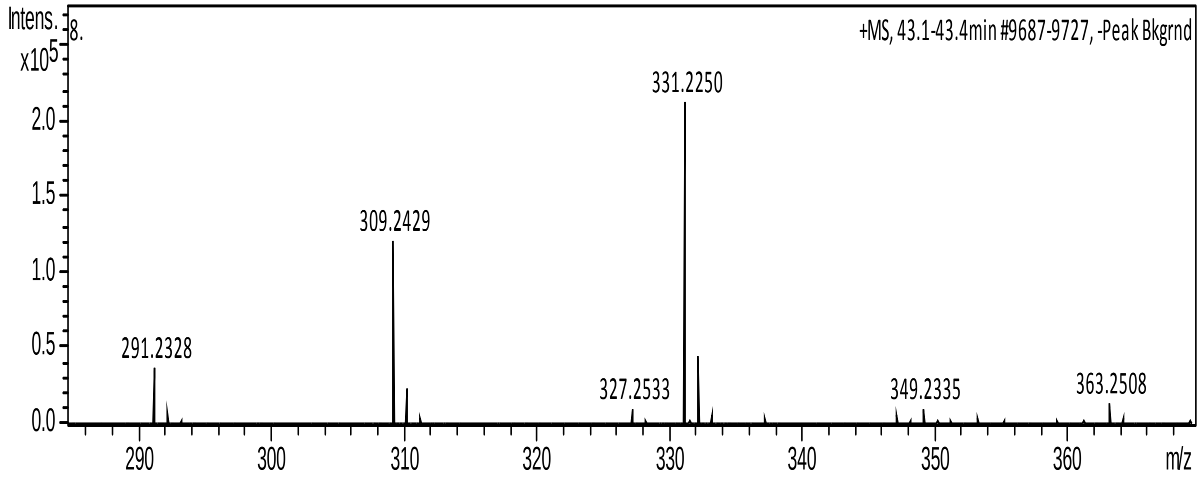

Supplementary Figure 4: HRESIMS spectrum (positive mode) of compound **1**

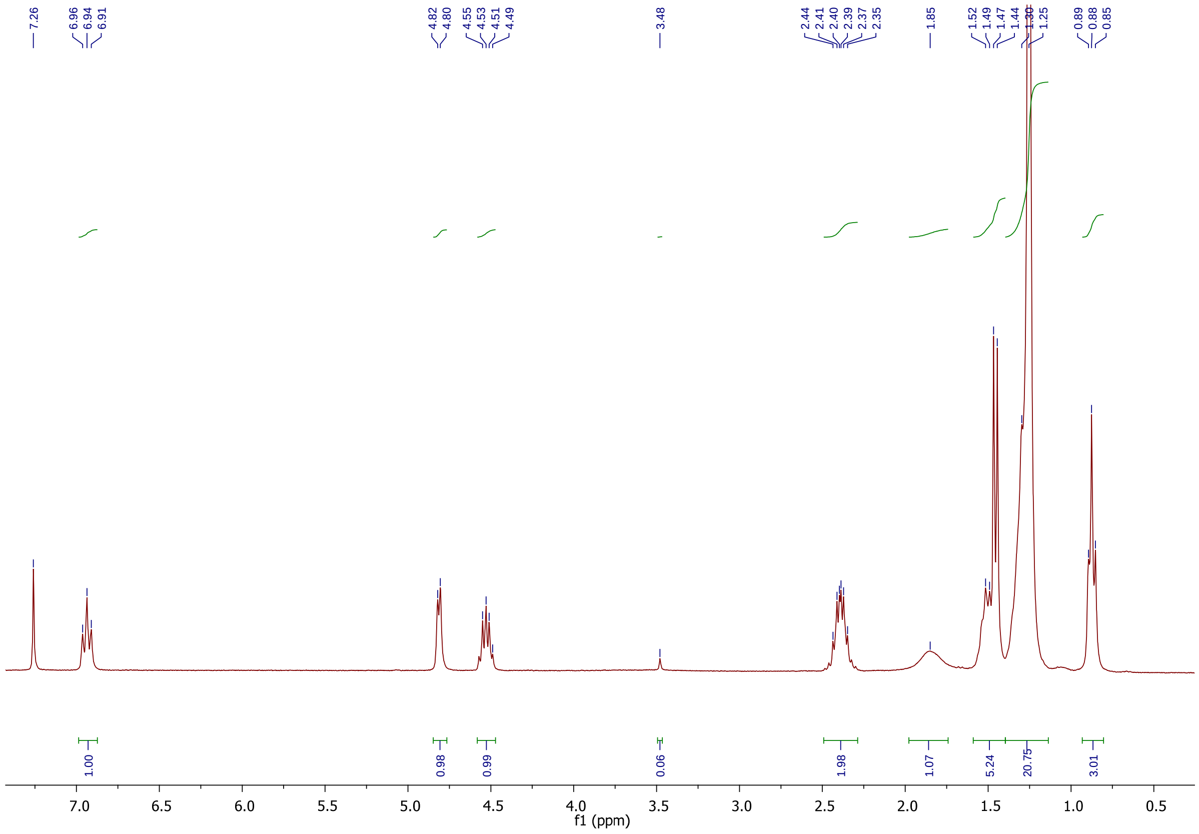

Supplementary Figure 5: ^{1}H NMR spectrum of compound **2** (300 MHz, $CDCl_3$)

EXCLI Journal 2020;19:323-333 – ISSN 1611-2156
Received: January 27, 2020, accepted: February 26, 2020, published: March 06, 2020

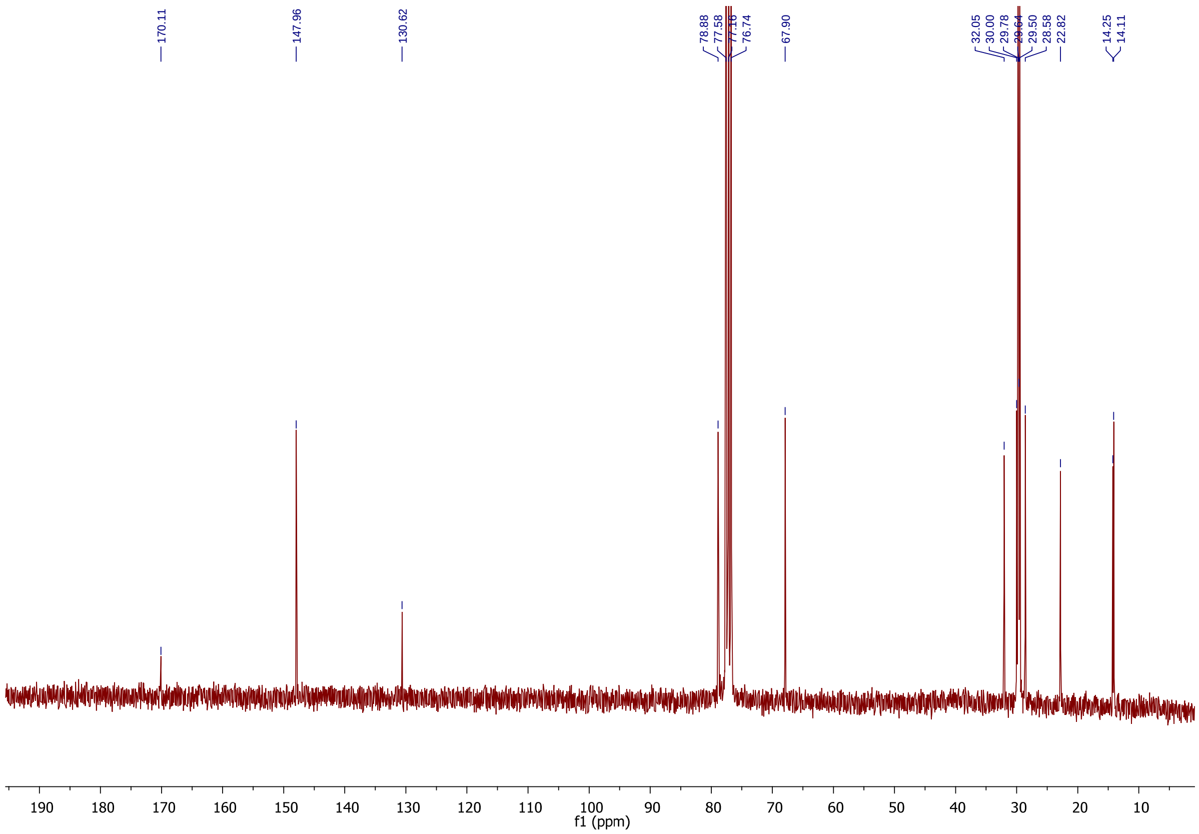

Supplementary Figure 6: ^{13}C NMR spectrum of compound **2** (75 MHz, $CDCl_3$)

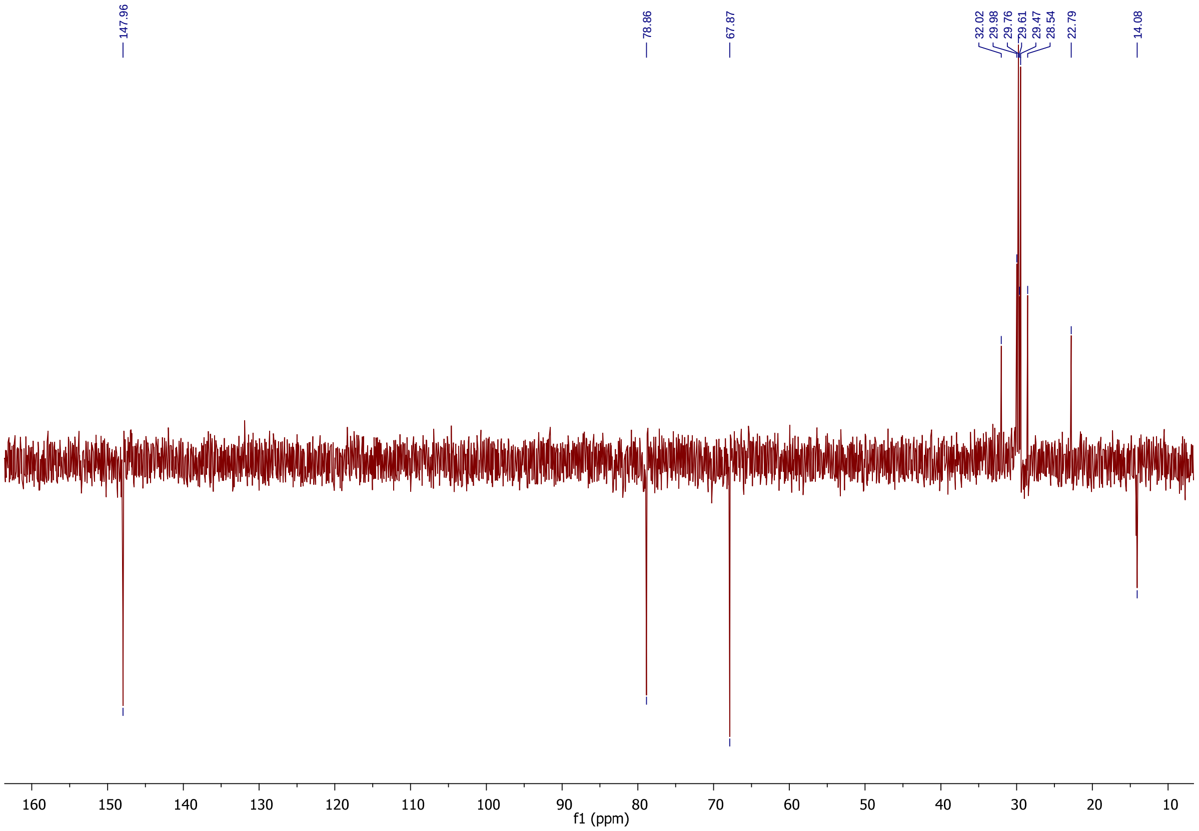

Supplementary Figure 7: DEPT-135 NMR spectrum of compound **2** (75 MHz, $CDCl_3$)

EXCLI Journal 2020;19:323-333 – ISSN 1611-2156
Received: January 27, 2020, accepted: February 26, 2020, published: March 06, 2020

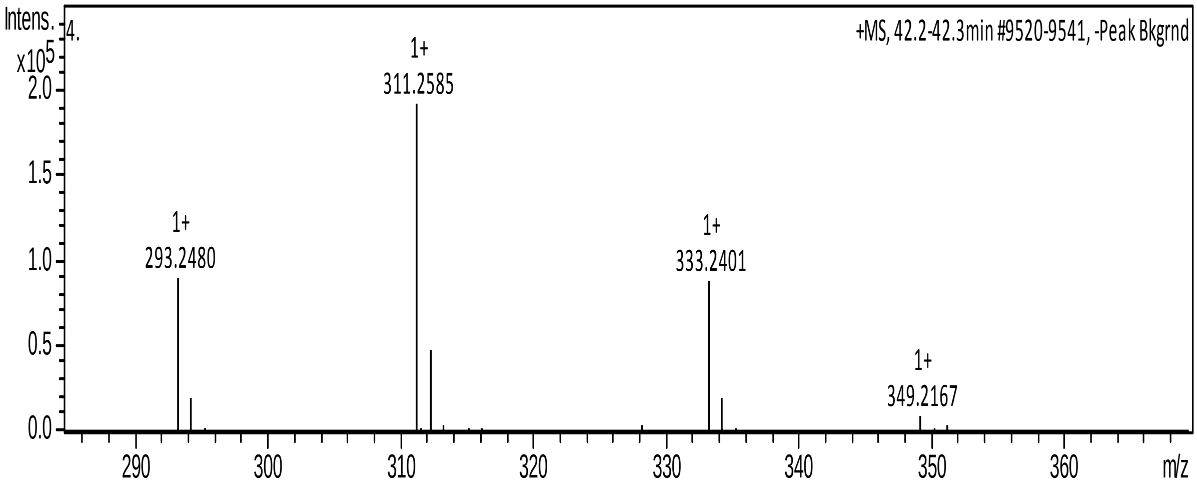

Supplementary Figure 8: HRESIMS spectrum (positive mode) of compound **2**

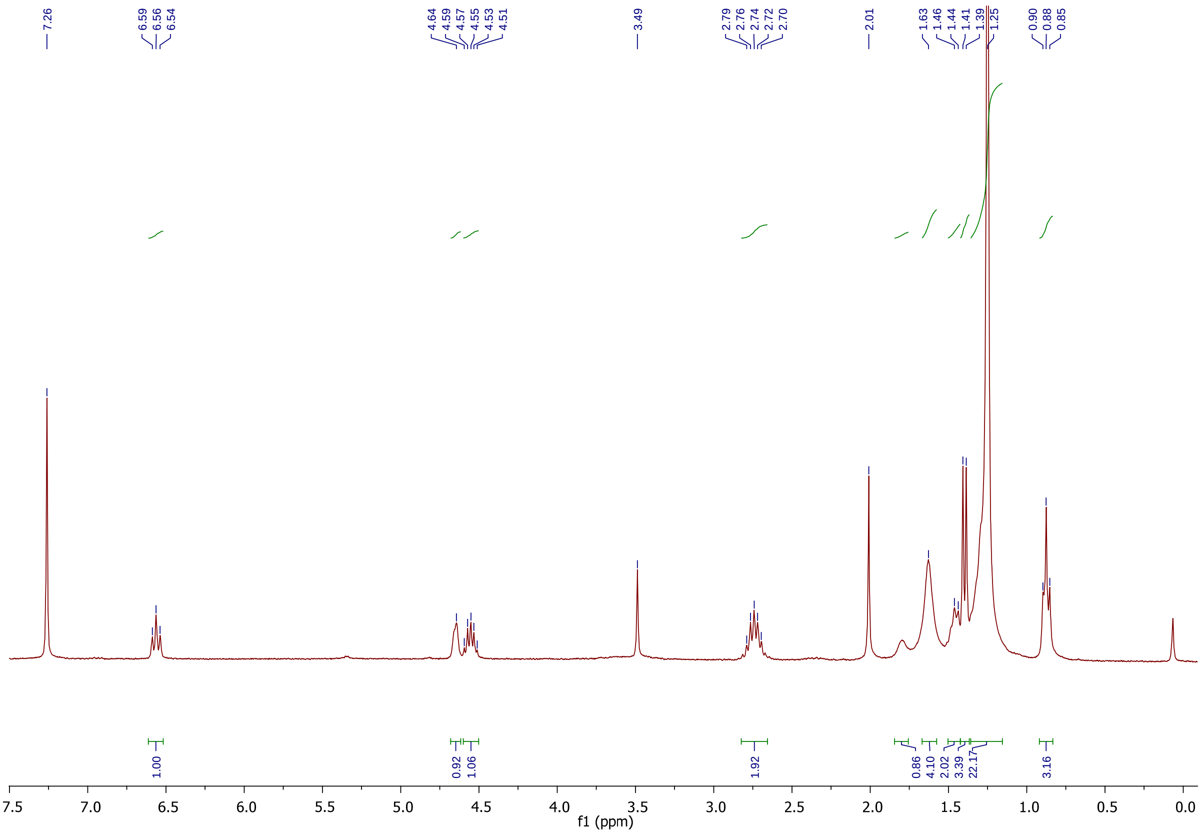

Supplementary Figure 9: ^{1}H NMR spectrum of compound **3** (300 MHz, $CDCl_3$)

EXCLI Journal 2020;19:323-333 – ISSN 1611-2156
Received: January 27, 2020, accepted: February 26, 2020, published: March 06, 2020

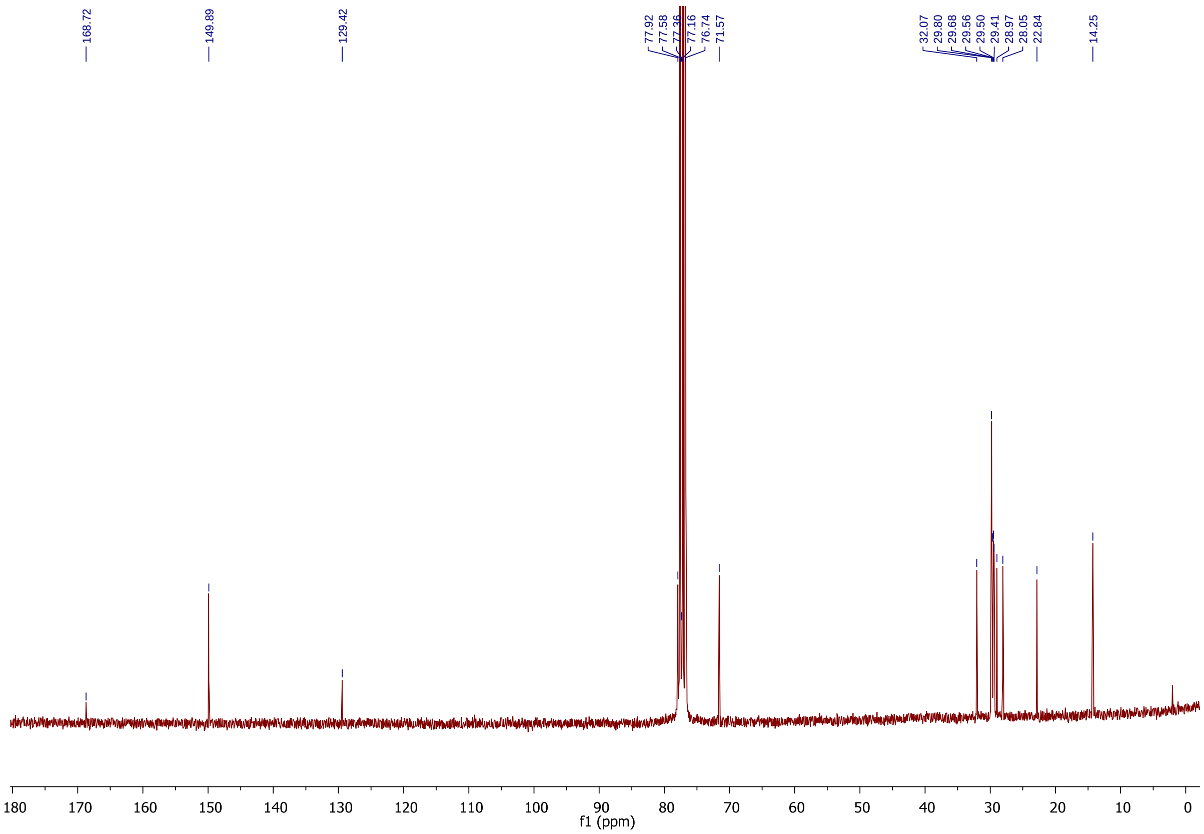

Supplementary Figure 10: ^{13}C NMR spectrum of compound **3** (75 MHz, $CDCl_3$)

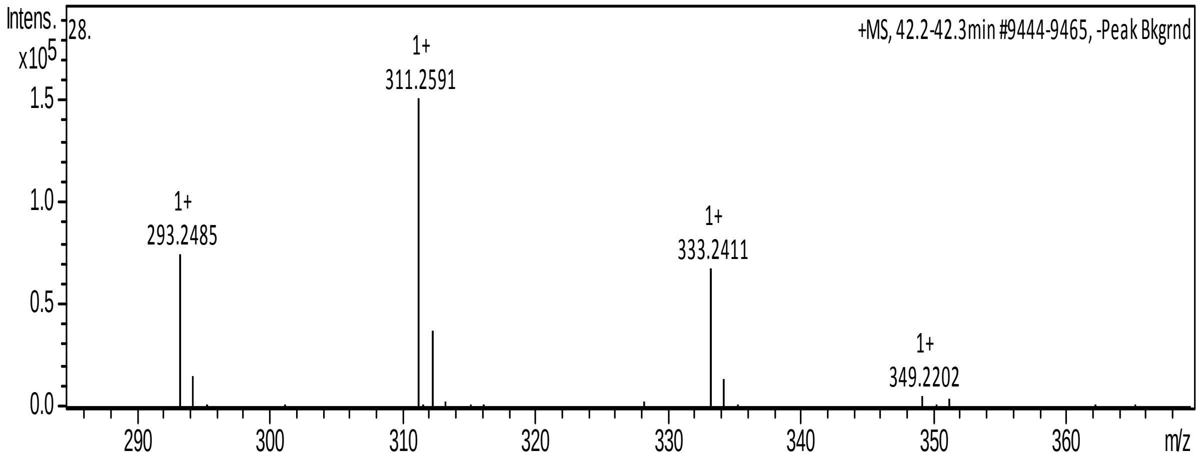

Supplementary Figure 11: HRESIMS spectrum (positive mode) of compound **3**

EXCLI Journal 2020;19:323-333 – ISSN 1611-2156
Received: January 27, 2020, accepted: February 26, 2020, published: March 06, 2020

Supplementary Table 1: Raw data of epimastigotes antiproliferative assay of isolated butanolides according to different time exposures. These data were used to calculate the IC_{50} values.

**Concentrations (µmol L^{-1})**	**Viability (%)**					
	**Isoobtusilactone A**					
	(24 h)			(72 h)		
0	100.0	100.0	100.0	100.0	100.0	100.0
1	100.0	100.0	100.0	100.0	100.0	100.0
2	81.77	84.6	87.43	100.0	100.0	56.63
4	46.79	45.56	46.79	31.91	29.08	23.72
8	36.45	35.95	35.46	5.05	6.54	4.07
16	22.16	22.16	29.55	2.23	2.71	1.33
	**Epilitsenolide C1**					
	(24 h)			(72 h)		
0	100.0	100.0	100.0	100.0	100.0	100.0
2	100.0	100.0	100.0	100.0	100.0	100.0
4	95.39	79.68	100	100	100	100
8	75.94	69.21	65.84	81.60	75.94	80.18
16	65.84	57.98	51.25	37.73	20.09	19.81
32	38.53	33.67	28.99	6.13	5.33	3.86
	**Benznidazole**					
	(24 h)			(72 h)		
0	100.0	100.0	100.0	100.0	100.0	100.0
5	83.75	85.42	84.58	96.95	93.9	100
10	84.42	76.04	80.23	76.5	81.9	79.2
20	60.30	77.05	68.67	41.5	47.2	44.35
40	15.74	34.50	25.12	11.2	12.6	16.3
80	4.69	5.02	4.85	7.2	8.5	7.1

Supplementary Table 2: Raw data of Vero cells cytotoxicity assay with the butanolides at 24 h of exposition. These data were used to calculate the CC_{50}/24 h values.

**Concentrations (µmol L^{-1})**	**Cell viability (%)**		
	**Isoobtusilactone A**		
0	100.0	100.0	100.0
12.5	100.0	100.0	100.0
25	98.28	98.72	104.5
50	90.43	87.74	90.06
100	92.69	89.12	90.62
200	25.41	20.14	22.02
400	10.66	8.22	8.59
	**Epilitsenolide C1**		
0	100.0	100.0	100.0
5	100	90.25	95.13
10	91.82	93.01	92.42
20	91.31	90.69	68.18
40	83.22	82.56	81.90
	**Benznidazole**		
0	100.0	100.0	100.0
211	100.0	100.0	100.0
422	100.0	100.0	100.0
845	60.43	60.34	57.16
1,691	39.58	36.97	37.85

EXCLI Journal 2020;19:323-333 – ISSN 1611-2156
Received: January 27, 2020, accepted: February 26, 2020, published: March 06, 2020

Supplementary Table 3: Epimastigotes concentration (10^5/mL) obtained from three replicates in the antiproliferative assay with **Isoobtusilactone A** according to the exposure time

**Concentrations (μmol L^{-1})**	Epimastigotes concentration (10^5/mL)								
	0 hours			24 hours			72 hours		
0	10.0	10.0	10.0	24.0	18.7	18.2	188.0	108.0	96.0
4	10.0	10.0	10.0	9.5	9.25	9.5	41.7	38.0	31.0
8	10.0	10.0	10.0	7.4	5.5	7.2	6.6	8.5	5.32
16	10.0	10.0	10.0	4.5	4.5	6.0	2.92	3.55	1.75
